# 

**Published:** 1917-01

**Authors:** 


Vol. XXXII JANUARY, 1917 No. 7

### TEXAS Medical Journal

